# Web‐based prognostic tools for oral tongue cancer: An analysis of online predictors

**DOI:** 10.1111/odi.15009

**Published:** 2024-07-05

**Authors:** Awais Wahab, Ibrahim O. Bello, Rasheed Omobolaji Alabi, Marco Mascitti, Giuseppe Troiano, Matti Mauramo, Ricardo D. Coletta, Tuula Salo, Alhadi Almangush

**Affiliations:** ^1^ Department of Pathology, Faculty of Medicine University of Helsinki, and Helsinki University Hospital (HUS) Helsinki Finland; ^2^ Department of Oral and Maxillofacial Diseases University of Helsinki Helsinki Finland; ^3^ Department of Oral Medicine and Diagnostic Sciences King Saud University Riyadh Saudi Arabia; ^4^ Research Program in Systems Oncology, Faculty of Medicine University of Helsinki Helsinki Finland; ^5^ Department of Industrial Digitalization, School of Technology and Innovations University of Vaasa Vaasa Finland; ^6^ Department of Clinical Specialistic and Dental Sciences Marche Polytechnic University Ancona Italy; ^7^ Department of Clinical and Experimental Medicine Foggia University Foggia Italy; ^8^ Department of Oral Diagnosis and Graduate Program in Oral Biology, School of Dentistry University of Campinas São Paulo Brazil; ^9^ Translational Immunology Research Program (TRIMM), Research Program Unit (RPU) University of Helsinki Helsinki Finland; ^10^ Department of Oncology, Comprehensive Cancer Center Helsinki University Hospital and University of Helsinki Helsinki Finland; ^11^ Research Unit of Population Health, and Medical Research Center Oulu University of Oulu and Oulu University Central Hospital Oulu Finland; ^12^ iCAN Digital Precision Cancer Medicine Flagship University of Helsinki Helsinki Finland; ^13^ University of Turku Institute of Biomedicine, Pathology Turku Finland; ^14^ Faculty of Dentistry Misurata University Misurata Libya

**Keywords:** Oral tongue squamous cell carcinoma, prediction, recurrence, survival, web‐based tools

## Abstract

**Background:**

Oral tongue squamous cell carcinoma (OTSCC) often presents with aggressive clinical behaviour that may require multimodality treatment based on reliable prognostication. We aimed to evaluate the prognostic ability of five online web‐based tools to predict the clinical behaviour of OTSCC resection and biopsy samples.

**Methods:**

A total of 135 OTSCC resection cases and 33 OTSCC biopsies were included to predict recurrence and survival. Area under the receiver operating characteristic curves (AUC), χ^2^ tests, and calibration plots constructed to estimate the prognostic power of each tool.

**Results:**

The tool entitled ‘*Prediction of risk of Locoregional Recurrences in Early OTSCC*’ presented an accuracy of 82%. The tool, ‘*Head & Neck Cancer Outcome Calculator*’ for 10‐year cancer‐related mortality had an accuracy 77% and AUC 0.858. The other tool entitled ‘*Cancer Survival Rates*’ for 5‐year mortality showed an accuracy of 74% and AUC of 0.723. For biopsy samples, ‘*Cancer Survival Prediction Calculators*’ predicted the recurrence free survival with an accuracy of 70%.

**Conclusions:**

Web‐based tools can aid in clinical decision making of OTSCC. Three of five online web‐based tools could predict recurrence risk and cancer‐related mortality in resected OTSCC and one tool could help in clinical decision making for biopsy samples.

## 1 INTRODUCTION

Oral squamous cell carcinoma (OSCC) is the 16th most common cancer, with 377,713 estimated new cases and 177,757 deaths worldwide in 2020 (Sung et al., 2021). OSCC is often associated with early recurrence, poor response to anti‐tumour therapies, and ultimately poor prognosis. Survival prognostication and treatment planning in OSCC are commonly evaluated with the TNM staging system. However, prognosis and response to treatment vary considerably within the same stage of OSCC (Panarese et al., 2019; Sawazaki‐Calone et al., 2015). Recently, there has been an increase particularly in the incidence of oral tongue squamous cell carcinoma (OTSCC), with 89,212 cases reported annually in 22 international cancer registries (Ng et al., 2017). OTSCC has an even lower survival rate than other subsites of the oral cavity, due to high loco‐regional recurrence rates after treatment (Farhood et al., 2019; Yang et al., 2018). A short recurrence interval is due to several factors, such as extracapsular spread, positive margin status and lympho‐vascular invasion (Weckx et al., 2019). Detection of early recurrence reduces mortality and significantly increases overall survival rates (Safi et al., 2017; Sundermann et al., 2018).

Although there has been progress in the diagnosis and treatment of OTSCC, improvements in survival rates remain modest (Chandler et al., 2011). Early‐stage OTSCC is often treated with surgical excision alone, although some cases can present with an aggressive behaviour. Management of such cases requires multimodality treatment, including neck dissection and radio‐, chemo‐ and immunotherapies (Alabi et al., 2020; Heikkinen et al., 2019). Tumour‐staging characteristics have been the basis of treatment decisions, but additional decision‐making tools could further stratify patients for optimal treatment selection (Montero et al., 2014).

Several studies have focused on the development of prognostic prediction models with incorporation of results retrieved from patient clinical data (Kosvyra et al., 2019). Web‐based prognostic tools that integrate various individual prognostic indicators of patients have been developed and are considered beneficial in clinical decision making for oral cancer patients (Xu et al., 2021). Each web‐based tool is constructed through a mathematical equation or an algorithm that is based on a group of known prognostic variables from a known data set to predict individual survival (Emerick et al., 2013; Xu et al., 2021). In this study, we sought to evaluate the prognostic power of five available online web‐based tools for prediction of recurrence rate and survival in OTSCC patients.

## 2 MATERIALS AND METHODS

This study included 135 OTSCC cases treated at the A.C. Camargo Cancer Center, São Paulo, Brazil (*n* = 77) and at Marche Polytechnic University, Ancona, Italy (*n* = 58). In addition, incisional biopsy samples of 33 patients treated for OTSCC were retrieved from Helsinki University Hospital, Finland.

The use of patient samples and the data enquiry were approved by the A.C. Camargo Cancer Center (São Paulo, Brazil) and the Brazilian Human Research Ethics Committee (CAAE: 55927322.0.0000.5418). Ethical approval for Italian cases was obtained from the institutional review board of Marche Polytechnic University, Italy (CERM 2019–308). Ethical approval for biopsy samples was granted from the local institutional review boards of the University of Helsinki, Finland, (HUS/44/2019).

### 2.1 Search criteria for web‐based tools

We conducted a PubMed search for peer‐reviewed publications using the following key terms: cancer, head and neck, oral cavity, prognosis, survival, prediction calculator, prognostic tool and nomogram. Web‐based tools were also identified through a manual search.

### 2.2 Description and clinicopathological parameters of the examined web‐based tools

A summary of the five web‐based tools is presented in Table 1. The tools marked as A to E are described briefly below:

- ‘*Oral Cavity Squamous Cell Carcinoma – Percentage of 5‐Year Locoregional Recurrence‐Free Survival*’ was developed by Gross et al. with a proportional hazard regression model by incorporating a training data set of 590 OSCC patients from the United States (Gross et al., 2008). This tool was designed to predict recurrence‐free survival and is one of the tools in the Cleveland Clinic Risk Calculator Library. This tool requires the following clinicopathological variables: gender, age, smoking history, surgical margin status, N classification, T stage, grade and primary tumour site (Gross et al., 2008). The tool is available at https://riskcalc.org/OralCancerOralCavitySquamousCellCarcinoma/
- ‘*Cancer Survival Prediction Calculators*’ was developed by Wang et al. to predict 5‐year locoregional recurrence‐free survival of OSCC patients with surgery alone or surgery along with radiotherapy. Different models were compared with the Akaike Information Criterion (AIC) and quantile–quantile (Q–Q) plots to identify the best performing model. This tool was developed with a log‐normal model from an oral cancer database in Brazil (Wang et al., 2010). This tool requires the following clinicopathological factors: age, sex, smoking history, tumour site, T stage, N stage, grade and tumour margin status (Wang et al., 2010).
  For HNC (head and neck cancer) conditional overall survival prediction, tool **B** was constructed from a Cox proportional hazards (CPH) semi‐parametric model with data from the surveillance, epidemiology and end results (SEER) database from patients diagnosed with HNC between 1995 and 2003. In this model, all covariates including patient and tumour characteristics were converted to binary variables, excluding age as continuous variable (Wang et al., 2011).
  To predict conditional survival (the time a patient has already survived since diagnosis and treatment of the cancer), this tool requires the following clinicopathological parameters: age, sex, origin (race), site, nodal stage extension, grade and months already survived (Wang et al., 2011). The tool is available at https://dmice.ohsu.edu/nomograms/headneck/oral.php
https://dmice.ohsu.edu/nomograms/headneck/headneck.php
- ‘*Head and Neck Cancer Outcome Calculator*’ is one of the tools (Nomograms) on CancerMath.net, which was developed by Emerick et al. at the Laboratory for Quantitative Medicine at Massachusetts General Hospital, Boston, MA, USA (Emerick et al., 2013). The nomogram is a binary‐biological model constructed from JavaScript, Hypertext Pre‐processor (PHP), and Hyper Text Markup Language (HTML), using XML/SWF charts v5.07 with Adobe Flash to train on the SEER 2009 data set of 17 cancer registries (Emerick et al., 2013). This model is based on correlation of cancer related mortality with parameters such as tumour size, number of nodes, site, age at diagnosis, race and tumour extension. It constitutes a series of equations to isolate impact of prognostic factors on patient outcome to estimate risk of death. It consists of the relationship between tumour size and risk of death (the *SizeOnly* equation) and the relationship between size, number of positive nodes and the risk of death (the *Size* + *Nodes* equation). Additional prognostic markers could be added by using *Size* + *Nodes* + *PrognosticMarkers* (SNAP) equation. This tool requires the following parameters: age, sex, origin (race), years since diagnosis, tumour site, tumour diameter, tumour extension, N stage, number of positive nodes, extracapsular spread and histological type of the tumour. All these factors have been related to the risk of death due to cancer. The tool is available at http://www.lifemath.net/cancer/headneck/outcome/index.php
- ‘C*ancer Survival Rates*’ was developed by Courage Health©2022 to predict survival rates in several types of cancers, including those of the oral cavity and oropharynx/tonsil. This tool was constructed based on a Cox proportional hazard model and was trained on the data set retrieved from National Cancer Institute SEER data registries between 2004 and 2017. The following parameters are required to predict cancer‐specific survival in the oral cavity: sex, age, stage and time since diagnosis. The tool is available at https://cancersurvivalrates.com/calculator.html?sex=M&age=65&stage=2&diagnosed=0&histology=scc‐and‐similar‐variants&type=oral‐cavity&years=5&role=doctor&chartSpan=5
- ‘*Prediction of Risk of Locoregional Recurrences in Early Oral Tongue Cancer*’ is the most recently developed tool by Alabi et al. This tool was constructed with artificial neural networks (ANNs) in a training data set of 311 patients from Finland and Brazil. MATLAB (R2018b version) was used to train and simulate ANN for classification. Web‐based prognostic estimator was developed with the Microsoft Azure machine learning studio (Azure, 2018) to predict prognosis of an individual case. The tool requires the following clinicopathological parameters: age, gender, T stage, grade, tumour budding, depth of invasion, worst pattern of invasion (WPOI), lymphocytic host response (LHR), perineural invasion (PNI), disease‐free months and follow‐up in months (Alabi et al., 2019). This tool is available at https://predictrecurrence.azurewebsites.net/

**TABLE 1:** Summary of web‐based tools

Web‐based tool	Author/Year	Cancer type in training data set	Tool construction	Training data set	Validation data set	Output
(A) Oral Cavity Squamous Cell Carcinoma – Percentage of 5‐Year Locoregional Recurrence‐Free Survival	Gross et al., 2008	OSCC	Proportional hazards regression model	590 patients with OSCC MSKCC	417 patients with OSCC HACC	Percentage of 5‐year locoregional recurrence free‐survival
(B) Cancer Survival Prediction Calculators						
Oral Cavity Post‐Op RT	Wang et al., 2010	OSCC	Log‐normal model	MSKCC and HACC	Bootstrapping method	5‐year locoregional recurrence‐free survival
Conditional Survival Prediction – Head and Neck cancer	Wang et al., 2011	HNC	Cox proportional hazards semi‐parametric model	SEER 1995–2003	Bootstrap correction with 100 resamples	Overall survival
(C) Head and Neck Cancer Outcome Calculator	Emerick et al., 2013	HNC	JavaScript, PHP, and HTML using XML/SWF Charts v5.07 with Adobe Flash	17SEER registries 2009	Massachusetts General Hospital 1362 patients	Percentage of 10‐year cancer mortality/Kaplan–Meier death rate percentage/Disease‐specific survival
(D) Cancer Survival Rates^a^	‐	SEER registries 2004–2017	Cox proportional hazard models	‐	‐	Cause‐specific survival
(E) Prediction of Risk of Locoregional Recurrences in Early Oral Tongue Cancer	Alabi et al., 2019	OTSCC	Artificial neural networks	311 patients with OTSCC, 1979–2009 Finland, Brazil	59 patients with OTSCC, 1998–2008, UOPECCAN Cancer Hospital, Brazil	Predicts risk for recurrence

Abbreviations: HACC Hospital do Cancer AC Camargo in São Paulo, Brazil; HNC head and neck cancer; MSKCC Memorial Sloan‐Kettering Cancer Center, USA; OSCC oral (cavity) squamous cell carcinoma; OTSCC oral tongue squamous cell carcinoma; RT Radiotherapy; SEER Surveillance, Epidemiology and End Results database.

^a^ Study not found for this tool.

### 2.3 Statistical methods

IBM SPSS Statistics for Windows version 25 (IBM Corp., Armonk, NY, USA) was used to determine the number and percentage distribution of clinicopathological characteristics in our data. The difference between actual and predicted value of a web‐based tool to evaluate prognostic power of each tool was analysed using receiver operating characteristic (ROC) analysis. The area under the ROC curve (AUC) was used to calculate the performance of each web‐based tool based on the predicted result of the input data. An AUC of 1.0 indicates perfect discrimination and an AUC of 0.5 indicates no discriminative performance of a web‐based tool. The AUC value of 0.7‐0.8 is an acceptable discrimination and AUC of 0.8‐0.9 indicates excellent discrimination. Model calibration was quantified by comparing observed and predicted values with χ^2^ tests to calculate the sensitivity, specificity and accuracy of each tool. The sensitivity, specificity and accuracy of each tool were calculated in the range from 0 to 100%. A measure of ≤50% was interpreted as non‐significant. Calibration plots were constructed using R studio package (Figure S1) and were followed according to the recommendations by ‘The Transparent Reporting of a multivariable prediction model for Individual Prognosis or Diagnosis’ (TRIPOD), (Collins et al., 2015).

## 3 RESULTS

### 3.1 Patient characteristics

Clinicopathological data of the 135 patients are summarized in Table 2. Median age at time of diagnosis was 61 (21–93) years. There were 87 (64.4%) male and 48 (35.6%) female patients. There were 29 (21.5%) well‐differentiated, 62 (45.9%) moderately differentiated and 44 (32.6%) poorly differentiated tumours. Forty‐two patients had T1N0M0 tumours and 93 had T2N0M0 tumours. Seventy‐five (55.6%) patients had free tumour surgical margins, while 60 (44.4%) patients had closed tumour surgical margins (Table 2). At the time of the last follow‐up, 39 patients (28.88%) had recurrence and 27 patients (20%) had died of cancer (Table 3).

**TABLE 2:** Clinicopathological characteristics of patients with OTSCC.

Clinicopathological characteristics	*n* (%)
Age (years), median (min‐max)	61 (21–93)
Gender	
Male	87 (64.4)
Female	48 (35.6)
Origin	
Caucasian	125 (92.5)
Non‐Caucasian	10 (7.5)
Grade	
I	29 (21.5)
II	62 (45.9)
III	44 (32.6)
T‐stage (Brazilian data: cT; Italian data: pT)	
1	42 (31.1)
2	93 (68.9)
Surgical Margin Status	
Free margin (>5 mm)	75 (55.6)
Closed margin (0.1–4.9 mm)	60 (44.4)
Smoking status of Brazilian data	77
Smokers	51 (66.24)
Non‐smokers	26 (33.76)
Characteristics of Italian data	58
Perineural Invasion	
Positive	12 (20.69)
Negative	46 (79.31)
Lymphocytic Host Response	
Type 1 (strong)	40 (68.97)
Type 2 (moderate)	17 (29.31)
Type 3 (weak)	1 (1.72)

*Note*: No patient had lymph‐node involvement at last follow‐up.

**TABLE 3:** Last follow‐up status of 135 patients.

Median (range) follow‐up in months	60 (6–258)
Treatment	
Surgery alone	106 (78.51%)
Surgery along with radiotherapy	29 (21.49%)
Number of recurrences	39 (28.88%)
Number of deceased patients	27 (20%)

*Note*: No patient had lymph‐node involvement at last follow‐up.

Among the 33 biopsy samples, 15 (45.5%) were well‐differentiated, 12 (36.4%) were moderately differentiated and 6 (18.2%) were poorly differentiated squamous cell carcinomas. Thirteen patients (39.4%) had T1, 11 (33.3%) had T2, 1 (3%) had T3 and 8 (24.3%) had T4 stage tumours (Table 4).

**TABLE 4:** Clinicopathological characteristics of OTSCC biopsy samples.

Clinicopathological characteristics	*n* (%)
Patient age (years), median (min‐max)	64 (27–91)
Patient gender	
Male	18 (54.5)
Female	15 (45.5)
Grade	
I	15 (45.5)
II	12 (36.4)
III	6 (18.2)
T‐stage	
1	13 (39.4)
2	11 (33.3)
3	1 (3.0)
4	8 (24.3)
Lymph node status	
N+	11 (33.33)
N0	17 (51.52)
Missing cases	5 (15.15)

*Note*: Median (min‐max) follow‐up in months = 35 (8–135). Recurrence at last follow‐up, *n* = 8. Patients deceased at last follow‐up, *n* = 19. Smoking status was unknown for biopsy samples.

### 3.2 Prediction of locoregional recurrence

#### 3.2.1 Prediction of recurrence‐free survival

**FIGURE 1:**
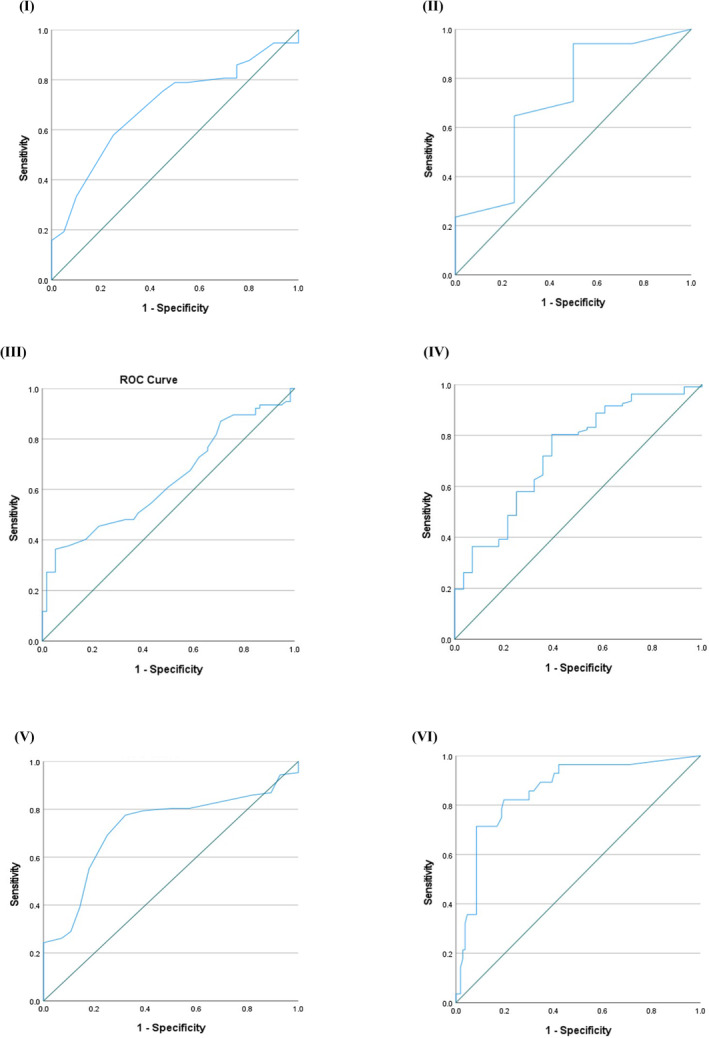
Receiver operating curves (ROC). (I) Tool **A**: Prediction performance for 5‐year locoregional recurrence‐free survival (AUC = 0.691); (II) Tool **B**: Prediction of recurrence‐free survival in biopsy samples (AUC = 0.713); (III) Tool **B**: Survival prediction in head and neck cancer (AUC = 0.636); (IV) Tool **C**: Survival prediction in head and neck cancer (AUC = 0.729); (V) Tool **D**: Prediction performance for cancer survival (AUC = 0.723); (VI) Tool **C**: Discriminatory power for cancer mortality (AUC = 0.858).

Two tools **A** and **B** were designed for prediction of recurrence‐free survival (Gross et al., 2008; Wang et al., 2010). Smoking status was unavailable for Italian cases, therefore only Brazilian data were evaluated for tool **A**. Fifty‐seven Brazilian patients had no recurrence until the last follow‐up. Tool **A** (Gross et al., 2008) was used to predict recurrence‐free survival and had an accuracy of 70%, AUC of 0.691 with a specificity of 78.9% (Table 5, Figure 1‐I).

**TABLE 5:** Evaluation of web‐based tools to predict prognosis in OTSCC.

Web‐based tools	Positive (%)	Negative (%)	Total	Area under ROC curve (95% CI)	*p*‐value	Number and sensitivity (%)	Number and Specificity (%)	Accuracy (%)
Prediction of locoregional recurrence‐free survival								
**(A)** Recurrence‐free survival^a^	57 (74.02)	20 (25.98)	77	0.691 (0.56–0.81)	0.011	9 (45)	45 (78.9)	70
**(B)** Recurrence‐free survival (Net benefit)	96 (71.11)	39 (28.89)	135	0.574 (0.46–0.68)	0.179	47 (49.0)	25 (64.1)	53
**(B)** Recurrence‐free survival (Surgery alone)	96 (71.11)	39 (28.89)	135	0.468 (0.36–0.57)	0.440	57 (59.4)	14 (35.9)	52
**(B)** Recurrence‐free survival (Surgery + RT)	96 (71.11)	39 (28.89)	135	0.508 (0.40–0.61)	0.949	52 (54.2)	14 (35.9)	48
Prediction of risk of recurrence								
**(E)** Risk of locoregional recurrence^b^	‐	‐	58	‐	‐	10 (52.6)	8 (97.4)	82
Prediction of survival								
**(B)** 5‐year survival OS	77 (57.03)	58 (42.97)	135	0.636 (0.54–0.72)	0.007	42 (54.5)	32 (55.2)	54
Prediction of cancer‐related mortality								
**(C)** Years mean survival DSS	107 (79.25)	28 (20.75)	135	**0.729 (0.62–0.83)**	**<0.001**	88 (82.2)	14 (50.0)	75
**(C)** 10‐year cancer mortality	28 (20.75)	107 (79.25)	135	**0.858 (0.78–0.93)**	**<0.001**	23 (82.1)	81 (75.7)	77
**(C)** Kaplan–Meier death rate	28 (20.75)	107 (79.25)	135	**0.765 (0.67–0.85)**	**<0.001**	23 (82.1)	77 (72.0)	74
**(D)** 5‐year survival rate DSS	107 (79.25)	28 (20.75)	135	**0.723 (0.62–0.82)**	**<0.001**	84 (78.5)	17 (60.7)	74

Abbreviations: DSS, Disease‐specific survival; OS, Overall survival; RT, Radiotherapy.

^a^ 77 cases were included as smoking status was unknown.

^b^ 58 cases were included to exclude data used in our previous study/AUC curve was not feasible.

Tool **B**; (Wang et al., 2010) for estimation of 5‐year locoregional recurrence‐free survival had an accuracy of 48%, AUC of 0.508 with a sensitivity of 54.2% (Table 5).

#### 3.2.2 Prediction of risk of recurrence

Tool **E** (Alabi et al., 2019) to predict recurrence risk had a high accuracy of 82%, sensitivity of 52.6% and specificity of 97.4%.

#### 3.2.3 For biopsy samples

Tool **B** (Wang et al., 2010) had an accuracy of 70%, AUC of 0.713 with a specificity of 83.3% (Table 6, Figure 1‐II).

**TABLE 6:** Evaluation of web‐based tools for biopsy samples of OTSCC.

Web‐based tools	Positive (%)	Negative (%)	Total	Area under ROC curve (95% CI)	*p‐*value	Number and sensitivity (%)	Number and specificity (%)	Accuracy (%)
Prediction of locoregional recurrence‐free survival								
**(B)** Recurrence‐free survival (Net benefit)^a^	19 (73.07)	7 (26.93)	26	0.594 (0.30–0.88)	0.470	2 (10.5)	5 (71.4)	26
**(B)** Recurrence‐free survival (Surgery alone)^a^	20 (74.07)	7 (25.93)	27	0.632 (0.38–0.88)	0.306	13 (65)	4 (57.1)	62
**(B)** Recurrence‐free survival (Surgery + RT)^a^	17 (80.95)	4 (19.05)	21	**0.713 (0.40–1.00)**	0.194	14 (66.7)	5 (83.3)	70
Prediction of survival								
**(B)** 5‐year survival	14 (42.42)	19 (57.58)	33	0.581 (0.37–0.78)	0.434	8 (57.1)	9 (47.4)	51
**(C)** Years mean survival	14 (42.42)	19 (57.58)	33	0.639 (0.44–0.83)	0.178	11 (78.6)	9 (47.4)	60
**(D)** 5‐year survival rate	14 (42.42)	19 (57.58)	33	0.656 (0.47–0.86)	0.094	10 (71.4)	12 (63.2)	66
**(D)** 10‐year survival rate	14 (42.42)	19 (57.58)	33	0.639 (0.43–0.84)	0.249	11 (78.6)	11 (57.9)	66

Abbreviation: RT, Radiotherapy.

^a^ Cases with missing data.

### 3.3 Prediction of survival

Seventy‐seven patients were alive at the last follow‐up. There were three tools for survival prediction.

Tool **B** (Wang et al., 2010) for prediction of 5‐year overall survival had an accuracy of 54%, AUC of 0.636 with a sensitivity of 54.5% (Table 5, Figure 1‐III).

The accuracy of tool **C** (Emerick et al., 2013) was 75%, AUC of tool for mean disease‐specific survival was 0.729 (Table 5; Figure 1‐IV), and had a sensitivity of 82.2% (Table 5).

Tool **D** for prognostication of 5‐year cancer‐specific survival had an AUC of 0.723 (95% confidence interval 0.62–0.82; *p* = 0.001) with a sensitivity of 78.5% and accuracy of 74% (Table 5; Figure 1‐V).

For biopsy samples: The best survival predictor for biopsy was tool **D** with an accuracy of 66%, and AUC of 0.656 with a sensitivity of 71.4% (Table 6).

### 3.4 Prediction of cancer‐related mortality

Twenty‐eight patients had died due to OTSCC at the last follow‐up. Tool **C** (Emerick et al., 2013) for prediction of 10‐year cancer‐related mortality had an accuracy of 77%, and prediction performance AUC of 0.858 with a sensitivity of 82.1% (Table 5; Figure 1‐VI).

## 4 DISCUSSION

This study sought to compare the prognostic performance of five web‐based tools in OTSCC. We found that three tools, **E**: *Prediction of Risk of Locoregional Recurrences in Early Oral Tongue Cancer*, **C**: *Head and Neck Cancer Outcome Calculator* and **D**: *Cancer Survival Rates* could predict risk of recurrence, cancer‐related mortality and cancer‐related survival in OTSCC, respectively. For biopsy samples, tool **B**: *Cancer Survival Prediction Calculators* could predict recurrence‐free survival.

The construction of predictive models by integrating clinical factors for decision making has been suggested in some cancers, such as mucosal melanoma (Xu et al., 2021), sarcoma (Eilber et al., 2004) and gastric carcinoma (Peeters et al., 2005). These nomograms have mostly been validated externally and are user‐friendly to guide clinicians in pre‐ and postoperative treatment planning. Although there are many prognostic tools to predict outcomes in HNC, none have been approved for clinical use. Most of the tools were designed using regression analysis techniques to stratify the risk group of HNC patients based on TNM stage and other pathological variables (Gross et al., 2008). It is rather challenging for clinicians to accurately assess HNC patients given the several relevant factors linked to prognosis. However, accurate diagnostic assessment could help clinicians to determine the most probable disease outcome and devise the treatment plan accordingly (Datema et al., 2013). Both patient‐related and tumour‐specific factors influence recurrence and survival predictions, including age, gender, race, immune status, size and grade of the tumour, in addition to other histological features (Emerick et al., 2013). While American Joint Committee on Cancer (AJCC) staging system aims to predict an increased likelihood of cancer‐related mortality, this staging is still not sufficiently accurate to determine prognosis. Therefore, mathematical models developed with a selected group of prognostic factors provide an alternative approach to better predict survival outcomes of cancer patients (Emerick et al., 2013).

Gross et al. performed an external validation of tool **A** (*Oral Cavity Squamous Cell Carcinoma – Percentage of 5‐Year Locoregional Recurrence‐Free Survival*) by comparing the predicted results with the actual outcome. For prediction of 5‐year locoregional recurrence‐free survival, this tool was externally validated using a series of 417 OSCC patients treated in Brazil. A concordance index (C‐index) of 0.693 was reported, which means that 69% of data pairs were correctly identified as locoregional recurrence (Gross et al., 2008). Our data in discriminatory analysis had an AUC of 0.691, and the model calibration showed a specificity of 78.9%, which indicates higher predictive power of the tool for cases having high probability of recurrence (Table 5; Figure 1‐I). Therefore, this tool could alert the clinicians to patients who are susceptible to recurrence. Although the smoking parameters were considered a least standardised variable, still its interaction with other variables was considered to make the model more predictable. Therefore, we consider that results could have been more promising if smoking parameter was not missing in our data. The clinicopathological parameters including T stage, grade and primary tumour site are known to have strong prognostic power for OTSCC (Farhood et al., 2019; Jardim et al., 2015; Lin et al., 2020; Niu et al., 2014). Furthermore, parameters in this tool such as advanced tumour stage and positive margin status have been correlated with an increased risk of locoregional recurrence to improve the predictive scores for this tool (Gross et al., 2008).

Wang et al. internally validated tool **B** (*Cancer Survival Prediction Calculators*) for the prediction of 5‐year locoregional recurrence‐free survival in OSCC using the Bootstrapping method for discrimination and calibration analysis with C‐index and calibration curve, respectively (Wang et al., 2010). The log‐normal model showed a C‐index of 0.66, which indicates good discrimination on internal validation. Our calibration analysis showed a specificity of 64.1% for prediction of 5‐year locoregional recurrence‐free survival for net benefit (Table 5). It indicates that this tool could better predict those cases which have a higher tendency to recurrence. For HNC prognostication, a Cox proportional hazards semiparametric model was evaluated with discrimination and calibration analysis using bootstrap correction with 100 resamples. Wang et al. internally validated the tool **B** and observed a C‐index of 0.70 for 5‐year conditional overall survival prediction. Their calibration curve also presented good agreement between observed and predicted outcome in HNC (Wang et al., 2011). Our analysis for this prognostic tool reported a sensitivity of 54.5% in the model calibration and had an AUC of 0.636 (Table 5; Figure 1‐III).

Tool **C** is among one of the web‐based calculators that can estimate survival in head and neck cancer, breast cancer, melanoma, renal cell carcinoma and colon cancer. (Emerick et al., 2013). To predict mortality rate, this tool was both internally and externally validated with SEER data and the Massachusetts General Hospital/Massachusetts Eye and Ear Infirmary (MGH/MEEI) data set, respectively (Emerick et al., 2013). The internal validation of the tool with the SEER data set presented a high C‐index value of 0.99 when SEER data were sorted into 4% lethality groupings as predicted by the SNAP model. It suggests that this model could accurately distinguish patients with a higher risk of cancer‐related mortality from patients with reduced risk of cancer‐related mortality (Emerick et al., 2013). The external validation of the tool was performed using the SNAP model with more than 100 groups of different combinations of tumour size, number of nodes and other prognostic factors. The investigators reported a high predictive index value of 0.975. Consistent with this, in discrimination analysis our data showed an AUC of 0.858, a sensitivity of 82.1%, and an accuracy of 77% for prediction of 10‐year cancer mortality (Table 5, Figure 1‐VI). The combination of incorporated clinicopathological parameters including tumour site, tumour diameter and number of positive nodes seem to be strong predictors for cancer related mortality in OTSCC (Farhood et al., 2019; Jardim et al., 2015; Li et al., 2021). The tool **C** with high AUC value and high sensitivity indicates its good predictive power for cases with higher chance of cancer‐related survival. Therefore, this tool could help clinicians in achieving a relatively accurate assessment of prognosis in OTSCC patients.

For prognostication in our cohort, tool **D** (C*ancer Survival Rates*) had a 5‐year cancer‐specific survival AUC of 0.723 with a sensitivity of 78.5% and specificity of 60.7% (Table 5; Figure 1‐V). The tool **D** has high sensitivity and AUC value, which indicates that it could correctly predict patients with a high expected survival rate. In this tool, age and stage are strong prognosticators of survival (Chen et al., 2020; Jardim et al., 2015).

The performance accuracy of ANN had been higher than logistic regression model and seemed to be an effective approach for predicting recurrences in OTSCC. The study for construction of tool **E** (*Prediction of Risk of Locoregional Recurrences in Early Oral Tongue Cancer*) reported a sensitivity and specificity of 71.2% and 98.9%, respectively (Alabi et al., 2019). This tool was validated in a cohort of 59 patients with OTSCC treated between 1998 and 2008 at UOPECCAN Cancer Hospital in Brazil and had a sensitivity of 78.9% (Alabi et al., 2019). Consistent with this, our cohort showed a specificity of 97.4% and an accuracy of 82% (Table 5), which indicates that this tool can prognosticate cases that are under less risk of recurrence and could also differentiate between high‐ and low‐recurrence risk patients. Age of the patient, tumour budding, depth of invasion, worst pattern of invasion and perineural invasion are parameters for this tool which have shown significant association with the recurrence in OTSCC (Alabi et al., 2019). Other markers such as gender, stage, grade, lymphocytic host response and follow‐up time serve as co‐founders to show independence of significant markers. Since this tool is constructed based only on oral tongue, therefore, its accuracy is deemed higher and could help clinicians in making a good prediction of OTSCC cases with high or low risk to determine the most appropriate treatment regime accordingly.

Recently, investigators have highlighted the role of biopsy specimen as advancing from being a mere diagnostic tool to becoming one for prognostication (Almangush et al., 2018; Dhanda et al., 2016; Jesinghaus et al., 2020; Seki et al., 2016). The utilization of biopsy samples as prognostic tools could help in minimizing unnecessary over and under treatment of patients (Bello et al., 2021). This is the main reason for including pre‐treatment biopsies in this study. In biopsy samples, tool **B** (*Cancer Survival Prediction Calculators*) had a high AUC of 0.713 and specificity of 83.3% for predicting 5‐year locoregional recurrence‐free survival for surgery along with radiotherapy. The high specificity of this tool shows that it can correctly predict true negative cases with a higher probability of recurrence. The clinicopathological factors in this tool including age, tumour site, T stage, grade and margin status are strong prognosticators for OTSCC (Chen et al., 2020; Farhood et al., 2019; Gross et al., 2008; Jardim et al., 2015; Lin et al., 2020; Niu et al., 2014; Park et al., 2022). Tool **D** (*Cancer Survival Rates*) in biopsy samples showed a sensitivity of 78.6% for prediction of 10‐year disease‐specific survival (Table 6). However, the number of biopsy samples was low, and a lot more cases should be analysed for proper statistical analyses.

The limitation in this study is the unavailability of some of the required parameters such as, smoking status and extracapsular spread for the analyses. The number of biopsy samples was too small for optimal statistical analyses and would require validation of the tools in a much larger cohort. Of note, each tool has been developed based on the TNM system available at the time of its development, making it difficult to generalize comparisons as the system has undergone significant changes over the years.

## 5 CONCLUSION

To the best of our knowledge, this is the first comparative analysis of the available online web‐based tools and nomograms for prognostication of OTSCC. Our results indicate that the web‐based tool for prediction of risk of locoregional recurrence, tool **E** ‘*Prediction of risk of Locoregional Recurrences in Early Oral Tongue Cancer*’ and the tools for prediction of cancer‐related mortality, **C** and **D**, ‘*Head and Neck Cancer Outcome Calculator*’ and ‘*Cancer Survival Rates*’ may be beneficial for this purpose. For biopsy samples, tool **B**, ‘*Cancer Survival Prediction Calculators*’ for predicting 5‐year locoregional recurrence‐free survival can be useful. Further studies in larger biopsy cohort with availability of all crucial parameters could help in further validation of this tool for its prognostic power.

These results underline the usefulness of the web‐based tools for clinicians to accurately make a predictive assessment of possible locoregional recurrence and cancer‐related mortality in OTSCC patients. To create the most clinically predictive instrument for both resected and biopsy OSCC samples, a novel prospective data from a broader OTSCC population should be tested to select the most accurate clinical and histological parameters to be included. Application of several of the most representative prognostic parameters in a web‐based tool would be a relevant step towards more accurate treatment planning for OTSCC patients.

## AUTHOR CONTRIBUTIONS

**Awais Wahab:** Conceptualization; investigation; formal analysis; writing – original draft; methodology; writing – review and editing; validation; software; data curation. **Ibrahim O. Bello:** Methodology; supervision; writing – review and editing; conceptualization; validation; writing – original draft. **Rasheed Omobolaji Alabi:** Conceptualization; software; methodology; investigation; writing – review and editing. **Marco Mascitti:** Software; methodology; investigation; formal analysis; validation; writing – review and editing; writing – original draft. **Giuseppe Troiano:** Conceptualization; methodology; validation; investigation; formal analysis; writing – original draft; writing – review and editing. **Matti Mauramo:** Conceptualization; software; methodology; data curation; resources; writing – review and editing; validation. **Ricardo D. Coletta:** Conceptualization; methodology; data curation; investigation; validation; formal analysis; supervision; visualization; project administration; writing – review and editing; writing – original draft. **Tuula Salo:** Conceptualization; software; investigation; formal analysis; funding acquisition; project administration; writing – original draft; writing – review and editing; resources; visualization; supervision; validation; data curation; methodology. **Alhadi Almangush:** Software; conceptualization; investigation; formal analysis; funding acquisition; project administration; writing – original draft; writing – review and editing; resources; visualization; validation; data curation; methodology; supervision.

## FUNDING INFORMATION

Sigrid Juselius Foundation; Helsinki University Central Hospital research funds, Finnish Dental Society Apollonia; University of Helsinki; Cancer Society of Finland.

## Supporting information


Figure S1.

## Data Availability

The data that support the findings of this study are available on request from the corresponding author. The data are not publicly available due to privacy or ethical restrictions.
