# Supplementary material for: Web‐based prognostic tools for oral tongue cancer: An analysis of online predictors
Source: Oral Dis. 2024 Jul 5;30(8):4867–77. doi: 10.1111/odi.15009 (PMC11610714; doi:10.1111/odi.15009)
Supplement: Supplementary file 1 — Figure S1. [file ODI-30-4867-s001.docx]

**
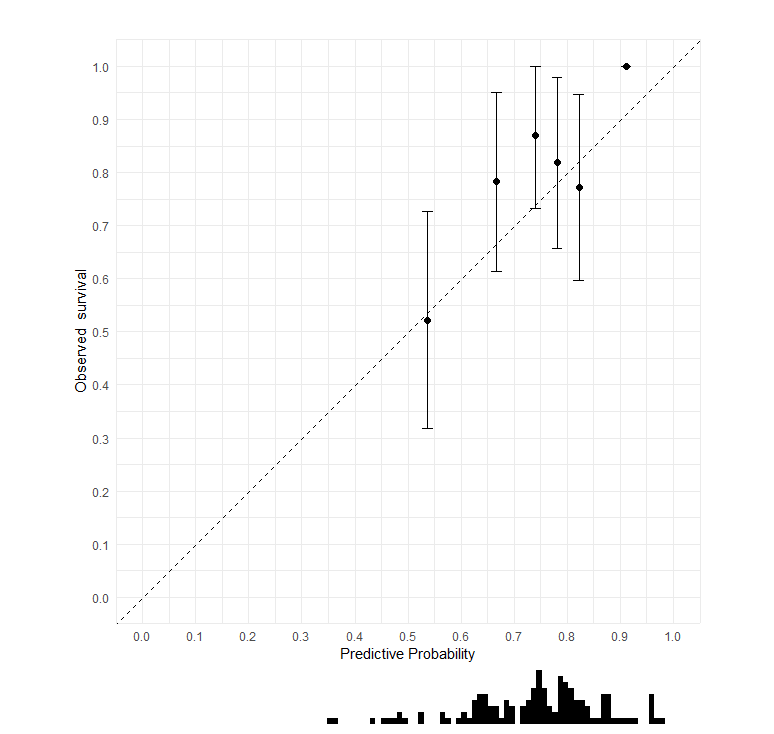
**
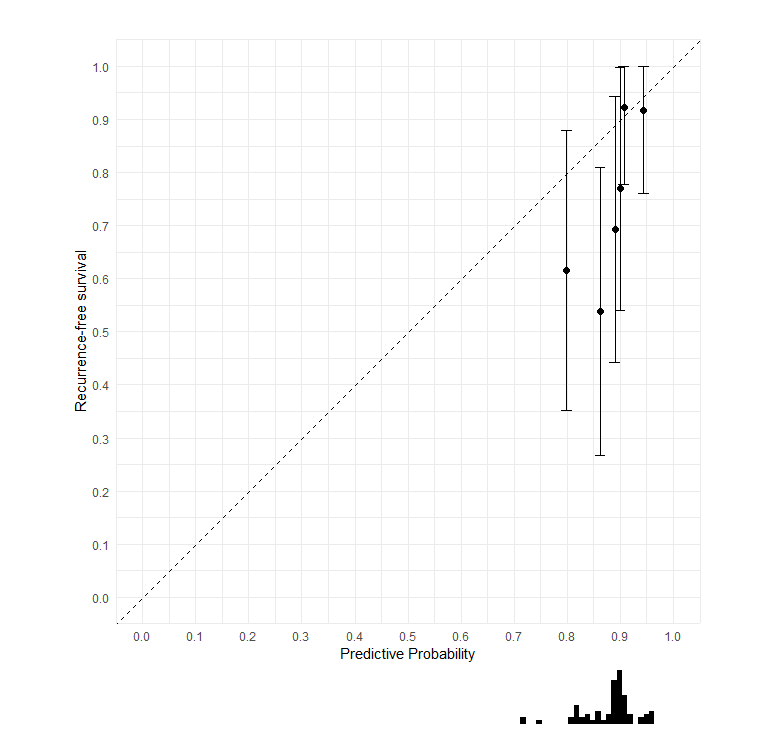
 **(I) (II)**

**Tool B:** Few groups show agreement between observed and predicted probability of survival.

**Tool A:** The calibration plot overestimates the predicted probability of recurrence-free survival.

**(III)** **(IV)**

**
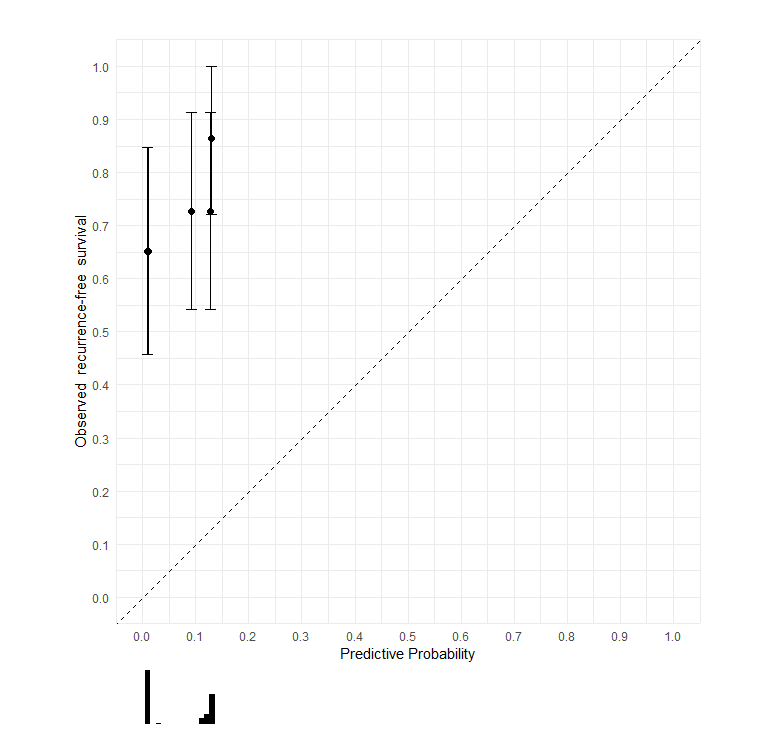

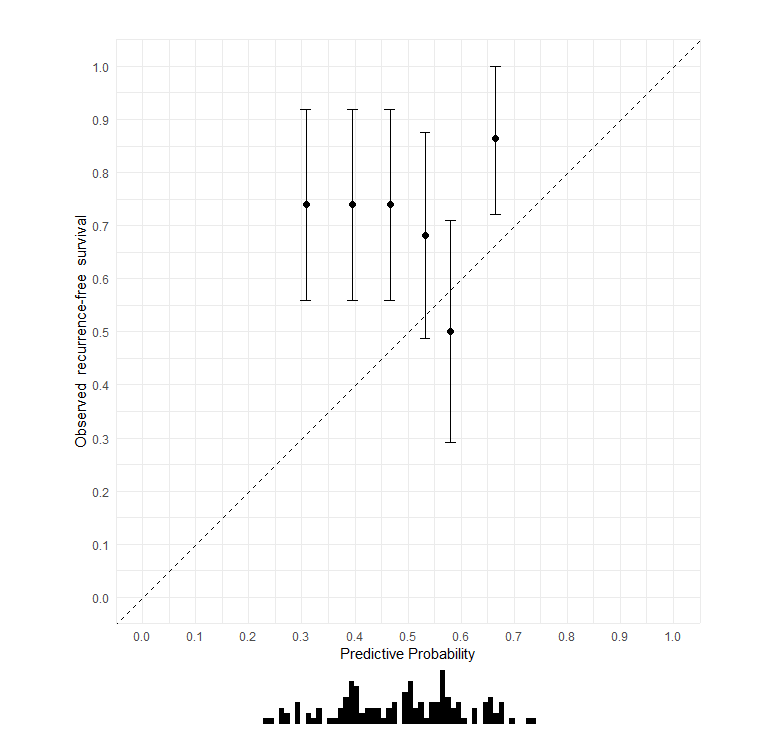
**

**Tool B:** The plot underestimates the predicted probability of recurrence-free survival.

**Tool B:** The plot underestimates predicted probability of recurrence-free survival.

**
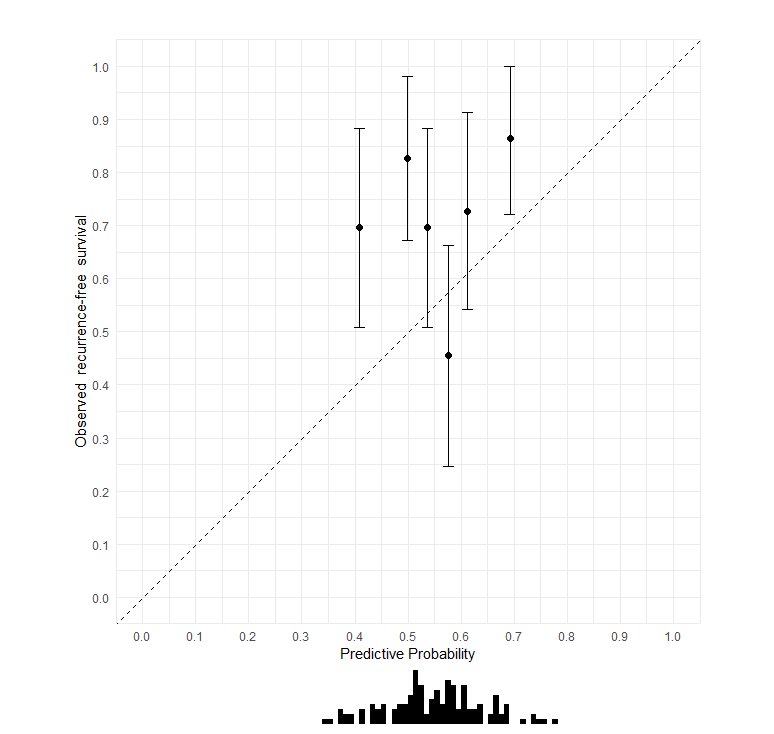

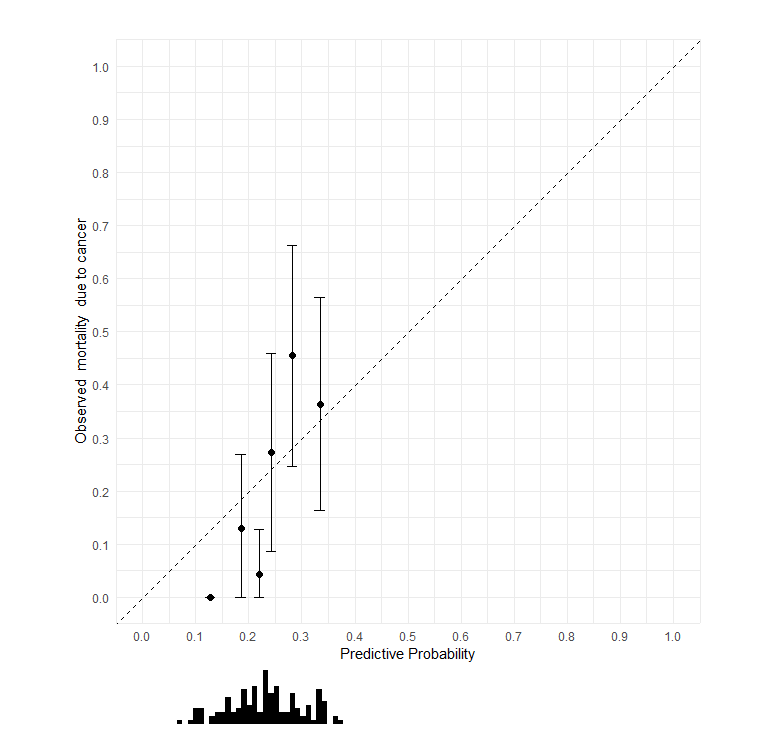
** **(V)** **(VI)**

**Tool C:** Some groups show agreement between predicted and observed probabilities. Few groups underestimate or overestimate the predicted risk of cancer-related mortality.

**Tool B:** The calibration plot underestimates predicted probability of recurrence-free survival.

**
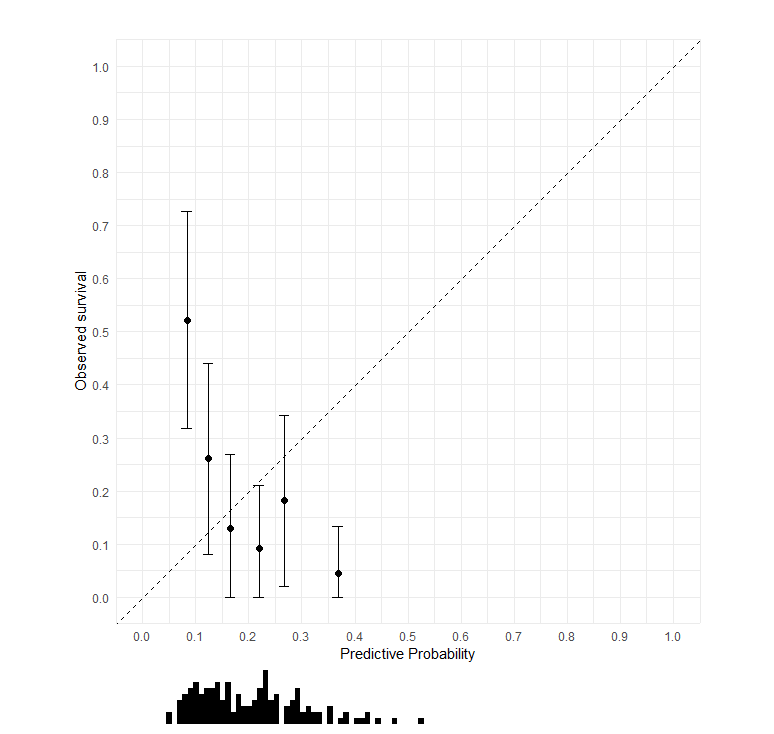

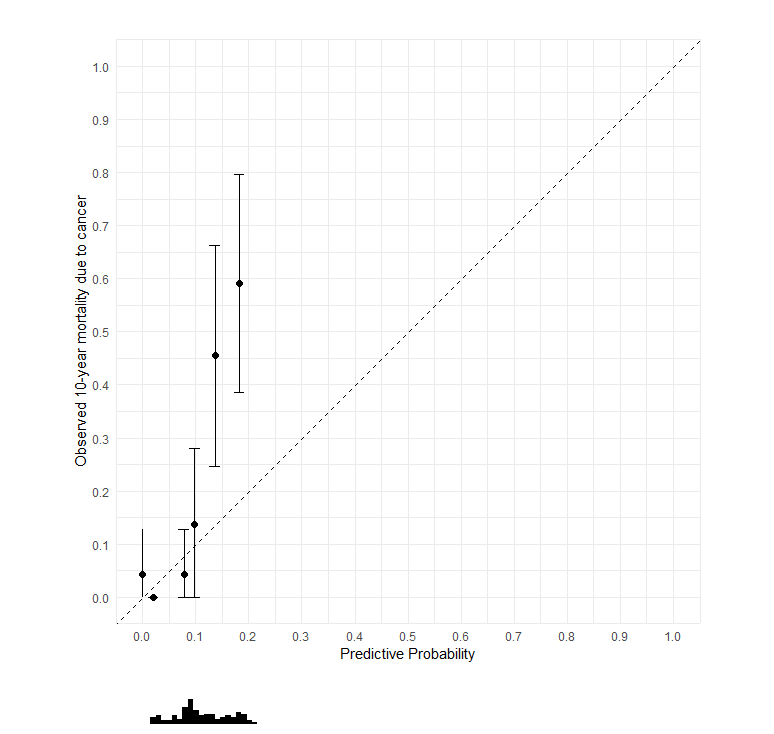
** **(VII)** **(VIII)**

**Tool C:** Some agreement between predicted and observed conditions among groups with lower mortality percentage, whereas the tool underestimates the risk of predicted mortality among high-percentage mortality groups.

**Tool C:** The calibration plot underestimates survival prediction in some groups.

**
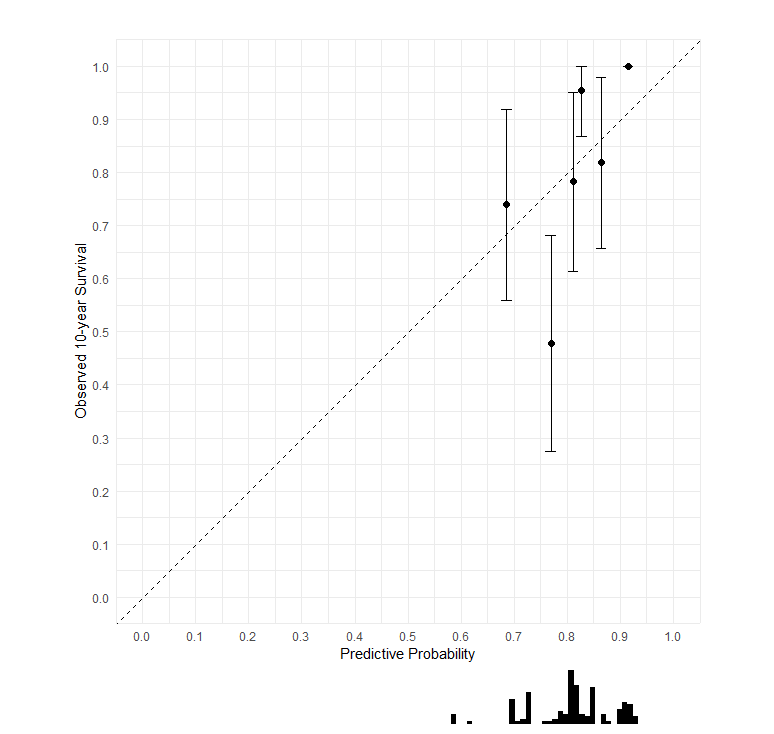

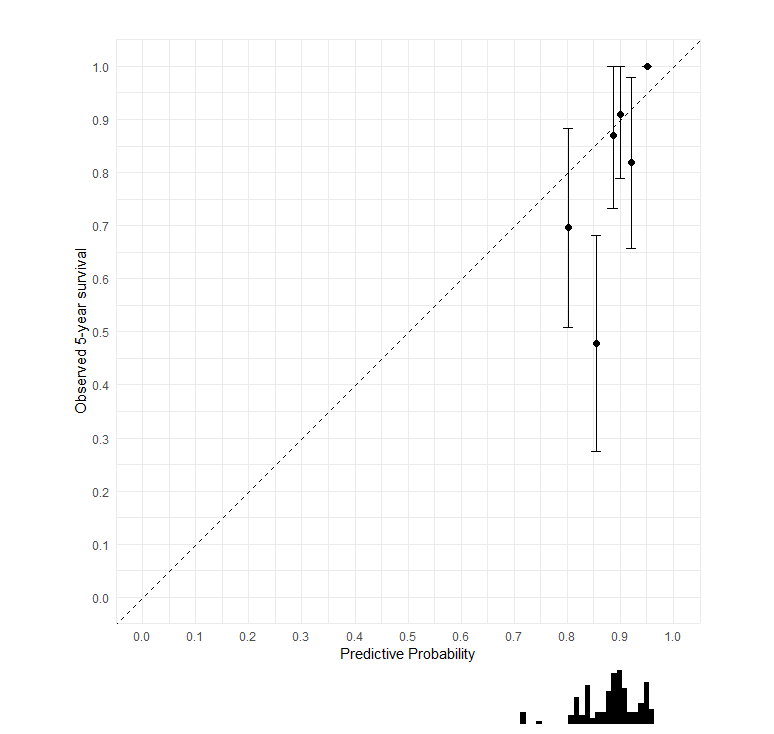
** **(IX)** **(X)**

**Tool D:** Few groups exhibit agreement and some underestimate predicted probabilities of 5-year survival.

**Tool D:** The calibration plot shows some groups in agreement, and few groups underestimate or overestimate predicted survival probabilities.
